# Hollow Mesoporous Silica@Zeolitic Imidazolate Framework Capsules and Their Applications for Gentamicin Delivery

**DOI:** 10.1155/2018/2160854

**Published:** 2018-04-05

**Authors:** Xiaoxiang Xu, Heyin Chen, Xia Wu, Sen Chen, Jing Qi, Zuhong He, Shengyu Zou, Le Xie, Kai Xu, Haitao Yuan, Yu Sun, Haoquan Zheng, Weijia Kong

**Affiliations:** ^1^Department of Otorhinolaryngology, Union Hospital, Tongji Medical College, Huazhong University of Science and Technology, Wuhan 430020, China; ^2^Key Laboratory of Applied Surface and Colloid Chemistry, Ministry of Education, School of Chemistry and Chemical Engineering, Shaanxi Normal University, Xi'an 710119, China

## Abstract

We have synthesized hollow mesoporous silica (HMS) at a zeolitic imidazolate framework (ZIF) capsule that can be used as a drug delivery system for gentamicin (GM). The GM is first loaded into HMS. Then, the outer surface of the GM/HMS is coated with uniformed ZIF nanoparticles (denoted as GM/HMS@ZIF). The GM/HMS@ZIF has been successfully prepared and acts as a capsule for GM. The GM/HMS@ZIF shows a good biocompatibility and a good cellular uptake in House Ear Institute-Organ of Corti 1 (HEI-OC1) cells. The GM is released slowly within 10 h under acidic conditions, which is used to simulate the pH of the endosome and lysosome compartments. The *in vivo* assay shows that the signal from fluorescein isothiocyanate (FITC) can be observed after 15 days, when the mice were injected with FITC/HMS@ZIF. This opens new opportunities to construct a delivery system for GM via one controlled low dose and sustained release for the therapy of Ménière's disease.

## 1. Introduction

Ménière's disease is a common inner ear disorder [1]. The clinical observation of Ménière's disease includes episodic vertigo, fluctuating hearing loss, tinnitus, and aural fullness, which negatively impacts the patient's life both physically and psychosocially. The gentamicin (GM) administration has been widely used for the treatment of Ménière's disease and has demonstrated to be effective in clinical applications. To avoid the systemic effect of aminoglycoside, Schuknecht developed a technique to deliver streptomycin intratympanically, which could control vertigo admirably [2]. Although intratympanic injection proves to be efficient, the high dose of the gentamicin in the local area of ears causes ototoxicity [3]. Despite that gentamicin is a very effective aminoglycoside, its potential ototoxicity which is of irreversible nature makes it a challenge and limits its application [4–13]. The use of low-dose and less frequent intratympanic gentamicin injection could solve this problem. More recently, controllable delivery systems for GM via sustained release have attracted many scientists' attentions. This kind of delivery system could deliver a precise and consistent amount of medicine to the round window due to continuous release of low-dose drug [14]. However, it is still challenging to construct a designable drug delivery system that can continuously release gentamicin and allow the control of the administration of the drug.

In the present study, the potential of nanomaterials as a neural interfacing material for drug release, neural repair, and regeneration has been widely explored [15–17]. More recently, metal-organic frameworks (MOFs) constructed by the coordination of metal ions or clusters with organic ligands have been developed [18–28]. MOFs have been used as a controlled delivery system for functional molecules, such as DNA, enzyme, fluorescein, and drugs [29–35]. Zeolitic imidazolate framework- (ZIF-) 8 is built from Zn ions and 2-methylimidazole (Hmim), which is a nontoxic and biocompatible ZIF [36–44]. However, the size of most drugs is larger than that of the pore opening of ZIF-8 (3.4 Å). It is difficult to load drugs by a postloading method. A one-pot synthetic route to encapsulate drugs in ZIF-8 nanoparticles has been developed [37]. A sustained release of drugs from the drug-loaded ZIF-8 has been achieved due to the decomposition of the ZIF framework under acidic conditions. However, the loading of drugs in ZIF is limited to 20 wt%. The use of high dose of ZIF carriers would give rise to the cytotoxicity due to the necrosis, which is caused by the nano-sized particle and unprogrammed cell death [34, 35, 45]. Therefore, a ZIF-based drug delivery system with a higher loading of drugs and a controlled release manner is still of high demand.

**Figure 1:**
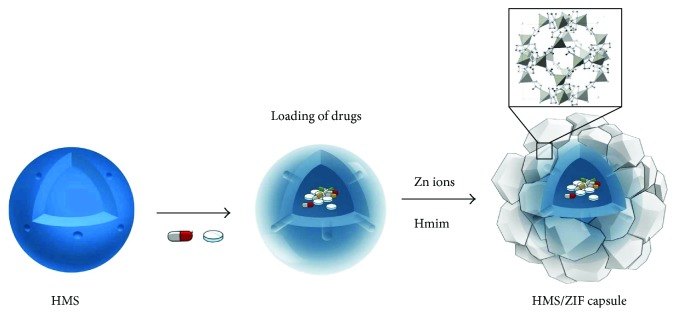
HMS/ZIF capsule for gentamicin.

Here, we have synthesized hollow mesoporous silica (HMS) at a ZIF capsule as a carrier for GM (Figure 1). The nanocarriers improve the existing treatment, since they can alter biodistribution profiles and pharmacokinetics. HMS has huge inner cavities and radially oriented mesochannels, which are useful for drug storage and delivery [46–50]. The mesochannels are perpendicular to the surface connecting the outer environment and the inner cavity. The GM is loaded in the inner cavities of the HMSs. The loading of GM can reach up to 38 wt% in GM/HMS@ZIF due to the big volume of the inner cavity. We demonstrate the sustained release of GM from the GM/HMS@ZIF capsule by *in vitro* and *in vivo* assays.

## 2. Materials and Methods

### 2.1. Synthesis of the HMSs

Typically, 60 mL of ethanol, 100 mL of H_2_O, and 2 mL of concentrated ammonia aqueous solution (25 wt% NH_3_) were added into a 250 mL flask. 0.3 g of CTAB was dissolved in ethanolic solution [51]. The mixture was then heated to 35°C, and tetraethyl orthosilicate (TEOS, 2 mL) was rapidly added under vigorous stirring. The solid mesoporous silica nanospheres were dispersed in distilled water (320 mL) at 100°C for 48 h and washed again with ethanol and dried under high vacuum. The surfactants were removed by calcination at 550°C for 6 h.

### 2.2. Synthesis of GM/HMS@ZIF Capsules

30 mg of HMS was suspended in 5 mL of ethanolic solution containing 70 mg GM. The suspension was conducted at 45°C under vacuum to remove the solvent. The obtained powder was dried by freezing-drying under vacuum. Then, 0.1 g of GM/HMS was added to 10 mL aqueous solution containing 1.89 g Hmim. Subsequently, a solution of 0.0975 g Zn(NO_3_)_2_ in 1 mL H_2_O was added. The mixture was stirred at room temperature for 5 min, followed by centrifugation and washing with deionized water. The GM/HMS@ZIF capsule was then obtained. The same synthesis approach was applied to prepare fluorescein isothiocyanate (FITC)/HMS@ZIF. Part of GM/HMS@ZIF was dissolved in HCl solution. The amount of GM in the supernatant was determined by high-performance liquid chromatography (HPLC). The detection of GM was carried out at 280 nm. The loading of GM was calculated as loading of GM (wt%) = *m*_GM_/*m*_total_.

### 2.3. Confocal Microscopy

House Ear Institute-Organ of Corti 1 (HEI-OC1) cells were seeded at a concentration of 1 × 10^6^ cells per well onto the surface of coverslips placed in plates and precultured for 24 h at 37°C. Then, the medium was removed, and fresh medium that contained FITC/HMS@ZIF (2 mg·L^−1^) was added. After 2, 8, or 24 h of incubation, the cells were washed twice with PBS and then fixed with 4% paraformaldehyde in PBS for 15 min at RT. The cells were then stained with 2.5 mg·L^−1^ of 4′,6-diamidino-2-phenylindole (DAPI) for 10 min and mounted with ProLong Gold antifade mounting medium. The stained samples were examined at excitation/emission wavelengths of 405/461 nm for DAPI and 490/530 nm for FITC.

### 2.4. MTT Assays in Breast Cancer

The Michigan Cancer Foundation- (MCF-) 7 cells were seeded at 1 × 10^5^ cells per well onto 96-well plates and were cultured in media containing 10% fetal calf serum. The suspensions of samples at various concentrations were added and cultivated at 37°C for 24 h. 10 *μ*L of MTT (3-(4,5,-dimethylthiazol-2-yl)-2,5-diphenyltetrazolium bromide) was added to each well after 24 h. The MCF-7 cells were further incubated for another 4 h. The MTT medium was removed, and then DMSO was added to each well. The absorbance at 570 nm was determined with a plate reader.

### 2.5. A Stepped Release of GM from GM/HMS@ZIF

10 mg of the GM/HMS@ZIF capsule was tested in a 20.0 mL buffer solution (pH 7.4) of 10% (*v*/*v*) FBS at 37°C. The pH of the solution was then adjusted to 5 with diluted HCl (0.6 M). The release percentages of GM were calculated according to the formula (release percentage (%) = mr/ml, in which mr is the amount of released GM while ml is the total amount of loaded GM). The amount of GM was determined by HPLC. When using FITC/HMS@ZIF, the amount of FITC was determined by a fluorescence spectrophotometer. The FITC was examined at excitation/emission wavelengths of 490/520 nm.

### 2.6. *In Vivo* Imaging of FITC/HMS@ZIF in a Rat Model

KM mice, 4-5 weeks old, were used. Mice were randomly assigned to three groups (*n* = 6): group 1 for PBS solutions as control, group 2 for free FITC solution, and group 3 for FITC/HMS@ZIF. 50 *μ*L of PBS solution, 50 *μ*L of free FITC (5 mg·mL^−1^), or 50 *μ*L of FITC/HMS@ZIF (10 mg·mL^−1^) was subcutaneously injected by postauricular hypodermic injections of mice. The images of mice were taken after treatment for 1, 3, 5, 8, 12, and 15 days. The mice were anesthetized by a mixed anesthesia of ketamine and chlorpromazine. The release of FITC in the ear after subcutaneous injection was monitored continuously by an in vivo imaging device. The samples were examined at excitation/emission wavelengths of 490/530 nm.

## 3. Results

**Figure 2:**
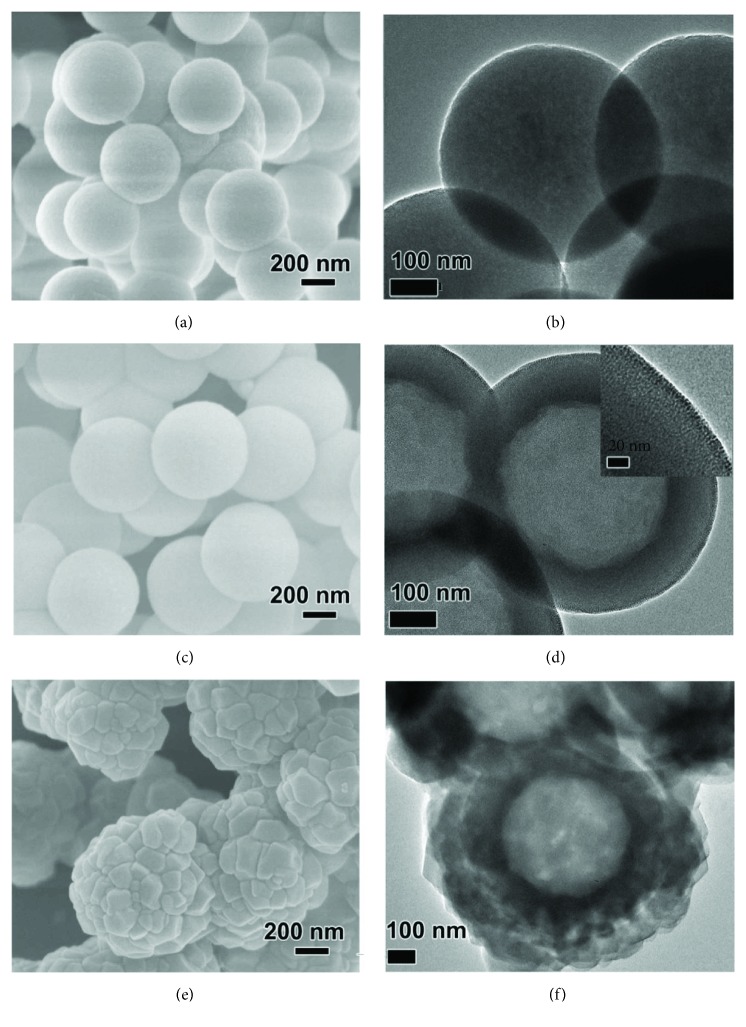
SEM images and TEM images of solid MS (a, b), HMS (c, d), and GM/HMS@ZIF (e, f), respectively.

**Figure 3:**
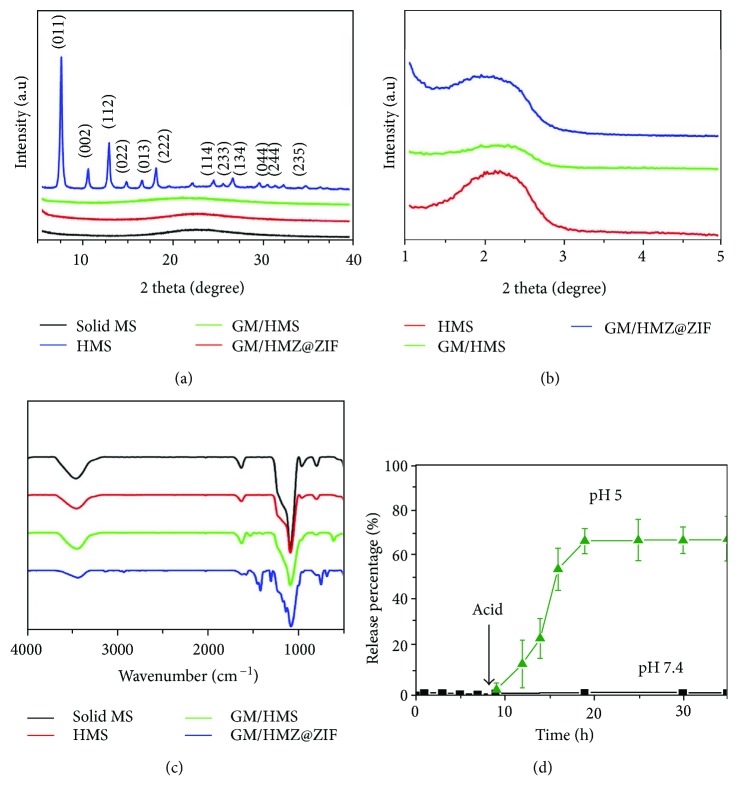
Wide-angle PXRD patterns (a), small-angle PXRD patterns (b), and FTIR spectra (c) of solid MS, HMS, GM/HMS, and GM/HMS@ZIF. (d) Release profiles of GM from GM/HMS@ZIF capsules by stepwise acidification.

The HMS was synthesized by two steps. The solid mesoporous silica (denoted as solid MS) was first generated by a surfactant assembly sol-gel process. A scanning electron microscopy (SEM) image showed that the solid MS had a particle size of 550 nm (Figures 2(a) and 2(b)). The solid MS was then incubated in H_2_O at 100°C for 48 h. The hydrothermal treatment changed the spontaneous morphology to the desired hollow structure, which was demonstrated by the difference between the hollow and the shell (Figures 2(c) and 2(d)). The mesopores in the shell enable the efficient mass transfer between the outside environment and the inner core (Figure 2(d), inset). As shown in Figure S1, the morphology of the GM-loaded HMS (denoted as GM/HMS) did not change. The powder X-ray diffraction (PXRD) patterns of solid MS, HMS, and GM/HMS showed a broad peak at 20°, which was attributed to the amorphous framework of the silica (Figures 3(a) and 3(b)). The Fourier-transform infrared spectroscopy (FTIR) showed the relative intensity of Si-O bending bands at 960 cm^−1^ in HMS and GM/HMS (Figure 3(c)). The infrared vibrations around 1350–1500 cm^−1^ were then assigned to the C-H vibrations of the GM in GM/HMS, demonstrating the successful loading of GM in HMS.

The mesopores in the shell are still accessible in GM/HMS. As shown in Figure 2(e), after coating ZIF-8 on the outer surface, HMS was completely covered by ZIF-8 nanoparticles. These nanoparticles present rhombic dodecahedral shapes with 50–200 nm in size. The hollow structure was not affected by the ZIF-8 coating. The sharp diffraction peaks of GM/HMS@ZIF fitted well with the previously reported peaks of ZIF-8, which implied the high crystallinity of ZIF-8. FTIR spectrum also demonstrated the formation of ZIF-8. N_2_ adsorption/desorption isotherms of GM/HMS@ZIF was shown in Figure S2. The capillary condensation in the range of p/p0 at 0.2-0.3, which can be attributed to the mesopores in the shell, disappeared. The mesopores were fully capped by ZIF-8 nanoparticles, and the GM/HMS@ZIF thus acted as a capsule and stored GM in the inner cavity. GM loading in the GM/HMS@ZIF was calculated to be 38 wt%. We tested the dynamic light scattering (DLS) of GM/HMS@ZIF (Figure S3). GM/HMS@ZIF had a narrow particle size distribution in the aqueous solution of 0.9% NaCl. These nanoparticles were highly dispersed, as a result of its high *ζ* potential of +30.1. There was no release of GM from GM/HMS@ZIF at pH 7.4 for 15 days, which demonstrated the safe storage of GM before passing the endosome and lysosome compartments (Figures 3(d) and S4) [52, 53].

**Figure 4:**
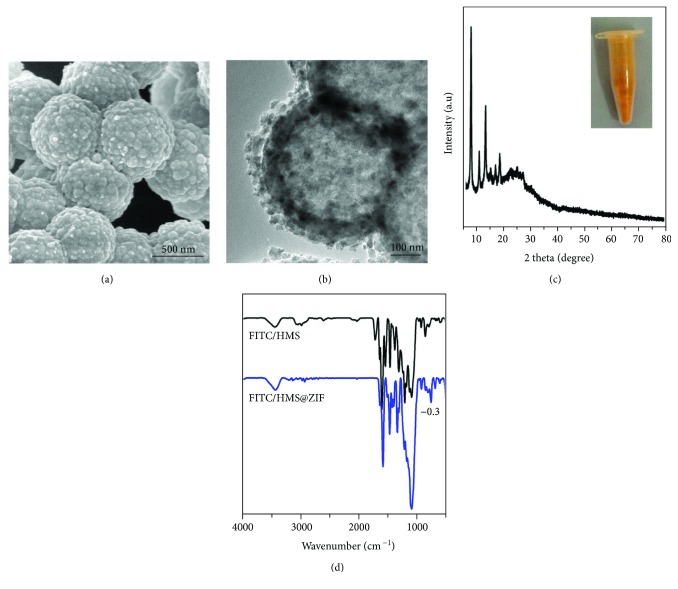
SEM image (a), TEM image (b), PXRD pattern (c), photograph (inset), and FTIR spectrum (d) of the FITC/HMS@ZIF capsule.

**Figure 5:**
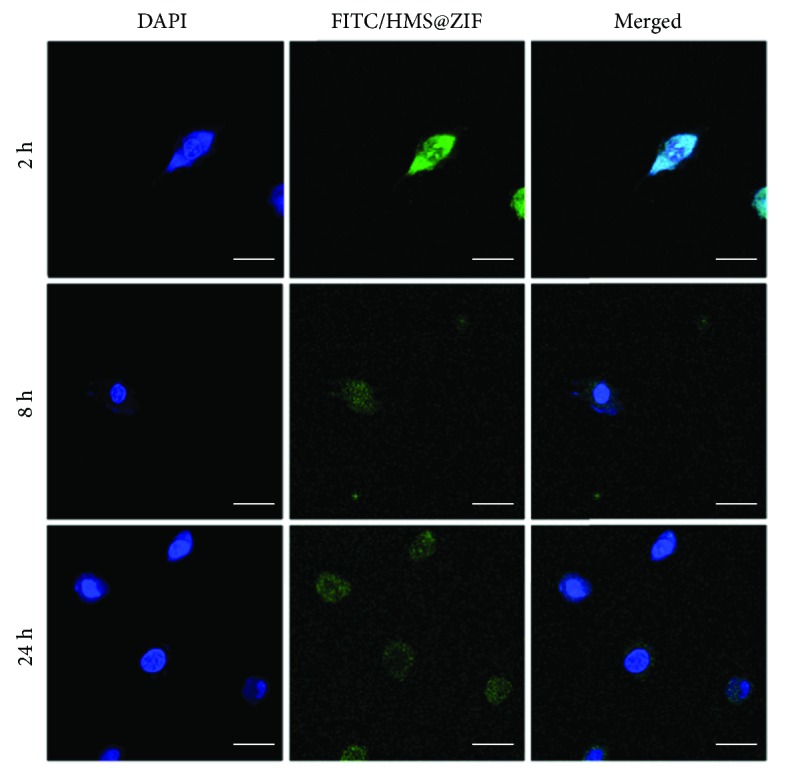
The cellular uptake study of the FITC/HMS@ZIF capsule in HEI-OC1 cells by the confocal microcopy images. The scale bar = 5 *μ*m.

We further studied the potential of the HMS@ZIF as a drug delivery system for GM. The cell assays showed that the viability of cells was higher than 95% after the treatment of MCF-7 cells with HMS@ZIF for 24 h. This result indicated that our material had a good biocompatibility (Figure S5). FITC was a modeling fluorescence and was used as a labelling agent in biomedicine. We chose FITC to replace GM for further investigation of the cellular uptake of our material. An SEM image showed that FITC/HMS@ZIF had a similar morphology to GM/HMS@ZIF (Figure 4(a)). As shown in Figure 4(b), the HMS was completely covered by nanocrystals. The PXRD demonstrated that these nanocrystals were ZIF nanocrystals (Figure 4(c)), while the FTIR spectra demonstrated the successful loading of FITC and the formation of ZIF in FITC/HMS@ZIF (Figure 4(d)). Confocal microscopy was then used to investigate the uptake of FITC/HMS@ZIF into HEI-OC1 cells (Figure 5). After the incubation with HEI-OC1 cells for 2, 8, and 24 h, the FITC/HMS@ZIF exhibited efficient intracellular uptake. The FITC/HMS@ZIF nanoparticles are located mainly in the cytoplasm and accumulated around the cell nuclei, which showed that the GM/HMS@ZIF nanocapsules had pass through the cell membrane. The transfer between the circulation in the bloodstream and the endosome and lysosome compartments (pH 5-6) was mimicked by a stepped release system. About 75% of GM were released slowly from the GM/HMS@ZIF within 10 h at pH 5.0. Therefore, a sustained release of GM from GM/HMS@ZIF can be achieved using this designable drug delivery system.

**Figure 6:**
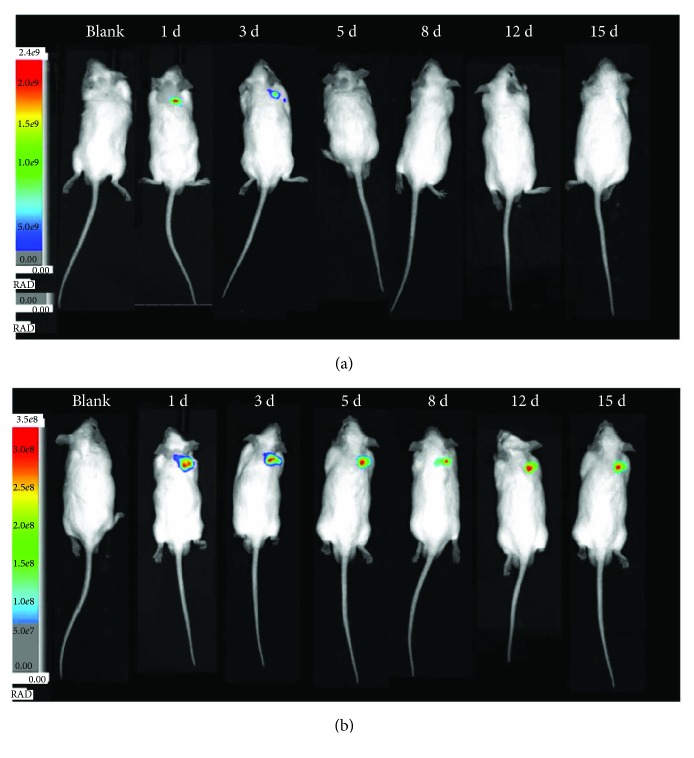
*In vivo* imaging of mice after administration of free FITC (a) and FITC/HMS@ZIF (b) at 1, 3, 5, 8, 12, and 15 days.

We took the *in vivo* images of mice after administration of free FITC and FITC/HMS@ZIF after several days (Figure 6). The mice were treated with free FITC or FITC/HMS@ZIF by postauricular hypodermic injections. When free FITC was used, no signal from FITC could be observed after 3 days. The signal with high intensity was observed from the mice injected with FITC/HMS@ZIF even after a long period of 15 days, demonstrating a continuous and sustained release of FITC form FITC/HMS@ZIF. Compared with free drug, the nanocarriers of HMS@ZIF may change both the pathways to the circulation due to the nanosize. Furthermore, the drug-eliminated half-life of GM might also change due to the freshly released GM at each subtle time period. Therefore, it is possible to construct an efficient delivery system with a controlled release manner. This novel delivery system for GM would make the treatment dosage of the GM precise and can be used to prevent the side effects of the GM, especially ototoxicity, during the treatments of Ménière's disease in clinical applications.

## 4. Discussion

We have synthesized HMS@ZIF capsule that can be used as controlled drug delivery system for GM. The GM has been first loaded into HMS. Then, the obtained GM/HMS has been coated with uniformed ZIF nanoparticles on the outer surface. The GM/HMS@ZIF has been successfully prepared and acts as a capsule. The GM/HMS@ZIF shows a good biocompatibility and a good cellular uptake in HEI-OC1 cells and is located in the cytoplasm. The GM is released slowly within 10 h under acidic conditions, which is used to simulate the pH of the endosome and lysosome compartments. The *in vivo* assay shows that the signal from FITC can be observed after 15 days from the mice that were injected with FITC/HMS@ZIF. This opens new opportunities to construct a delivery system for GM via one controlled low dose and sustained release for the therapy of Ménière's disease.

## Supplementary Materials

Supplementary Materials Figure S1: the HRTEM image of HMS (a) and TEM image of GM/HMS (b). Figure S2: N_2_ adsorption/desorption isotherms of HMS and GM/HMS@ZIF. Figure S3: size distributions of GM/HMS@ZIF in 0.9% NaCl solution. Figure S4: release profiles of GM from GM/HMS@ZIF capsules at pH 7.4 for 15 days. Figure S5: the function of mitochondria in the MCF-7 cells treated for 24 h.

## References

[1] da Costa S. S., de Sousa L. C. A., de Toledo Piza M. R. (2002). Meniere’s disease: overview, epidemiology, and natural history. *Otolaryngologic Clinics of North America*.

[2] Schuknecht H. F. (1956). Ablation therapy for the relief of Meniere’s disease. *The Laryngoscope*.

[3] Sam G., Chung D. W., van der Hoeven R., Verweij S., Becker M. (2016). The effect of intratympanic gentamicin for treatment of Ménière’s disease on lower frequency hearing. *International Journal of Clinical Pharmacy*.

[4] Ni W., Zeng S., Li W. (2016). Wnt activation followed by Notch inhibition promotes mitotic hair cell regeneration in the postnatal mouse cochlea. *Oncotarget*.

[5] He Z., Guo L., Shu Y. (2017). Autophagy protects auditory hair cells against neomycin-induced damage. *Autophagy*.

[6] Draz E. I., Abdin A. A., Sarhan N. I., Gabr T. A. (2015). Neurotrophic and antioxidant effects of silymarin comparable to 4-methylcatechol in protection against gentamicin-induced ototoxicity in guinea pigs. *Pharmacological Reports*.

[7] Cheng C., Guo L., Lu L. (2017). Characterization of the transcriptomes of Lgr5+ hair cell progenitors and Lgr5- supporting cells in the mouse cochlea. *Frontiers in Molecular Neuroscience*.

[8] Yu X., Liu W., Fan Z. (2017). c-Myb knockdown increases the neomycin-induced damage to hair-cell-like HEI-OC1 cells in vitro. *Scientific Reports*.

[9] Guan M., Fang Q., He Z. (2016). Inhibition of ARC decreases the survival of HEI-OC-1 cells after neomycin damage *in vitro*. *Oncotarget*.

[10] Waqas M., Zhang S., He Z., Tang M., Chai R. (2016). Role of Wnt and Notch signaling in regulating hair cell regeneration in the cochlea. *Frontiers of Medicine*.

[11] He Z., Sun S., Waqas M. (2016). Reduced TRMU expression increases the sensitivity of hair-cell-like HEI-OC-1 cells to neomycin damage in vitro. *Scientific Reports*.

[12] Liu L., Chen Y., Qi J. (2016). Wnt activation protects against neomycin-induced hair cell damage in the mouse cochlea. *Cell Death & Disease*.

[13] Sun S., Sun M., Zhang Y. (2014). *In vivo* overexpression of X-linked inhibitor of apoptosis protein protects against neomycin-induced hair cell loss in the apical turn of the cochlea during the ototoxic-sensitive period. *Frontiers in Cellular Neuroscience*.

[14] Liu B., Leng Y.-m., Shi H. (2015). Modified titration intratympanic gentamicin injection for unilateral intractable Ménière’s disease. *Journal of Huazhong University of Science and Technology [Medical Sciences]*.

[15] Guo R., Zhang S., Xiao M. (2016). Accelerating bioelectric functional development of neural stem cells by graphene coupling: implications for neural interfacing with conductive materials. *Biomaterials*.

[16] He Z., Zhang S., Song Q. (2016). The structural development of primary cultured hippocampal neurons on a graphene substrate. *Colloids and Surfaces B: Biointerfaces*.

[17] Zha Y., Chai R., Song Q. (2016). Characterization and toxicological effects of three-dimensional graphene foams in rats in vivo. *Journal of Nanoparticle Research*.

[18] Ding N., Li H., Feng X. (2016). Partitioning MOF-5 into confined and hydrophobic compartments for carbon capture under humid conditions. *Journal of the American Chemical Society*.

[19] Bloch E. D., Queen W. L., Krishna R., Zadrozny J. M., Brown C. M., Long J. R. (2012). Hydrocarbon separations in a metal-organic framework with open iron(II) coordination sites. *Science*.

[20] Rosi N. L., Eckert J., Eddaoudi M. (2003). Hydrogen storage in microporous metal-organic frameworks. *Science*.

[21] Li J.-R., Sculley J., Zhou H.-C. (2011). Metal–organic frameworks for separations. *Chemical Reviews*.

[22] Lee J., Farha O. K., Roberts J., Scheidt K. A., Nguyen S. T., Hupp J. T. (2009). Metal–organic framework materials as catalysts. *Chemical Society Reviews*.

[23] Tingting W., Yanyuan J., Qiang C. (2016). A new luminescent metal-organic framework for selective sensing of nitroaromatic explosives. *Science China Chemistry*.

[24] Allendorf M. D., Bauer C. A., Bhakta R. K., Houk R. J. T. (2009). Luminescent metal–organic frameworks. *Chemical Society Reviews*.

[25] Patel H. A., Islamoglu T., Liu Z. (2017). Noninvasive substitution of K^+^ sites in cyclodextrin metal–organic frameworks by Li^+^ ions. *Journal of the American Chemical Society*.

[26] Li Z., Peters A. W., Bernales V. (2017). Metal–organic framework supported cobalt catalysts for the oxidative dehydrogenation of propane at low temperature. *ACS Central Science*.

[27] Li P., Modica J. A., Howarth A. J. (2016). Toward design rules for enzyme immobilization in hierarchical mesoporous metal-organic frameworks. *Chem*.

[28] Shang L., Yu Y., Gao W. (2018). Bio-inspired anisotropic wettability surfaces from dynamic ferrofluid assembled templates. *Advanced Functional Materials*.

[29] Wu M.-X., Yang Y.-W. (2017). Metal–organic framework (MOF)-based drug/cargo delivery and cancer therapy. *Advanced Materials*.

[30] Horcajada P., Gref R., Baati T. (2011). Metal–organic frameworks in biomedicine. *Chemical Reviews*.

[31] Liang K., Ricco R., Doherty C. M. (2015). Biomimetic mineralization of metal-organic frameworks as protective coatings for biomacromolecules. *Nature Communications*.

[32] Deng J., Wang K., Wang M., Yu P., Mao L. (2017). Mitochondria targeted nanoscale zeolitic imidazole framework-90 for ATP imaging in live cells. *Journal of the American Chemical Society*.

[33] Guan J., Hu Y., Wang Y. (2017). Controlled encapsulation of functional organic molecules within metal–organic frameworks: in situ crystalline structure transformation. *Advanced Materials*.

[34] Della Rocca J., Liu D., Lin W. (2011). Nanoscale metal–organic frameworks for biomedical imaging and drug delivery. *Accounts of Chemical Research*.

[35] Horcajada P., Chalati T., Serre C. (2010). Porous metal–organic-framework nanoscale carriers as a potential platform for drug delivery and imaging. *Nature Materials*.

[36] Ren H., Zhang L., An J. (2014). Polyacrylic acid@zeolitic imidazolate framework-8 nanoparticles with ultrahigh drug loading capability for pH-sensitive drug release. *Chemical Communications*.

[37] Zhuang J., Kuo C.-H., Chou L.-Y., Liu D.-Y., Weerapana E., Tsung C.-K. (2014). Optimized metal–organic-framework nanospheres for drug delivery: evaluation of small-molecule encapsulation. *ACS Nano*.

[38] Huang X.-C., Lin Y.-Y., Zhang J.-P., Chen X.-M. (2006). Ligand-directed strategy for zeolite-type metal–organic frameworks: zinc(II) imidazolates with unusual zeolitic topologies. *Angewandte Chemie International Edition*.

[39] Park K. S., Ni Z., Cote A. P. (2006). Exceptional chemical and thermal stability of zeolitic imidazolate frameworks. *Proceedings of the National Academy of Sciences of the United States of America*.

[40] Sun C.-Y., Qin C., Wang X.-L. (2012). Zeolitic imidazolate framework-8 as efficient pH-sensitive drug delivery vehicle. *Dalton Transactions*.

[41] Röder R., Preiß T., Hirschle P. (2017). Multifunctional nanoparticles by coordinative self-assembly of His-tagged units with metal–organic frameworks. *Journal of the American Chemical Society*.

[42] Wuttke S., Lismont M., Escudero A., Rungtaweevoranit B., Parak W. J. (2017). Positioning metal-organic framework nanoparticles within the context of drug delivery – a comparison with mesoporous silica nanoparticles and dendrimers. *Biomaterials*.

[43] Levine D. J., Runčevski T., Kapelewski M. T. (2016). Olsalazine-based metal–organic frameworks as biocompatible platforms for H_2_ adsorption and drug delivery. *Journal of the American Chemical Society*.

[44] Zheng X., Wang L., Pei Q., He S., Liu S., Xie Z. (2017). Metal–organic framework@porous organic polymer nanocomposite for photodynamic therapy. *Chemistry of Materials*.

[45] Zheng H., Zhang Y., Liu L. (2016). One-pot synthesis of metal–organic frameworks with encapsulated target molecules and their applications for controlled drug delivery. *Journal of the American Chemical Society*.

[46] Teng Z., Su X., Zheng Y. (2015). A facile multi-interface transformation approach to monodisperse multiple-shelled periodic mesoporous organosilica hollow spheres. *Journal of the American Chemical Society*.

[47] Chen Y., Chen H., Guo L. (2010). Hollow/rattle-type mesoporous nanostructures by a structural difference-based selective etching strategy. *ACS Nano*.

[48] Wu M., Meng Q., Chen Y. (2016). Large pore-sized hollow mesoporous organosilica for redox-responsive gene delivery and synergistic cancer chemotherapy. *Advanced Materials*.

[49] Lu N., Huang P., Fan W. (2017). Tri-stimuli-responsive biodegradable theranostics for mild hyperthermia enhanced chemotherapy. *Biomaterials*.

[50] Zhang J., Weng L., Su X. (2018). Cisplatin and doxorubicin high-loaded nanodrug based on biocompatible thioether- and ethane-bridged hollow mesoporous organosilica nanoparticles. *Journal of Colloid and Interface Science*.

[51] Teng Z., Su X., Zheng Y. (2013). Mesoporous silica hollow spheres with ordered radial mesochannels by a spontaneous self-transformation approach. *Chemistry of Materials*.

[52] Zheng H., Xing L., Cao Y., Che S. (2013). Coordination bonding based pH-responsive drug delivery systems. *Coordination Chemistry Reviews*.

[53] Xing L., Zheng H., Cao Y., Che S. (2012). Coordination polymer coated mesoporous silica nanoparticles for pH-responsive drug release. *Advanced Materials*.

