# Supplementary material for: Hollow Mesoporous Silica@Zeolitic Imidazolate Framework Capsules and Their Applications for Gentamicin Delivery
Source: Neural Plast. 2018 Apr 5;2018:2160854. doi: 10.1155/2018/2160854 (PMC5907525; doi:10.1155/2018/2160854)
Supplement: Supplementary Materials — Figure S1: the HRTEM image of HMS (a) and TEM image of GM/HMS (b). Figure S2: N2 adsorption/desorption isotherms of HMS and GM/HMS@ZIF. Figure S3: size distributions of GM/HMS@ZIF in 0.9% NaCl solution. Figure S4: release profiles of GM from GM/HMS@ZIF capsules at pH 7.4 for 15 days. Figure S5: the function of mitochondria in the MCF-7 cells treated for 24 h. [file 2160854.f1.pdf]

Supporting Information for

Hollow Mesoporous Silica@Zeolitic Imidazolate Framework Capsules and Their Applications for Gentamycin Delivery

Xiaoxiang Xu,[a] Heyin Chen,[b] Xia Wu,[a] Sen Chen,[a] Jing Qi,[b] Zuhong He,[a] Shengyu Zou,[a] Le Xie,[a] Kai Xu,[a] Haitao Yuan,[b] Yu Sun,[a,] Haoquan Zheng,[b,]* Weijia Kong[a,]**

a. Department of otorhinolaryngology, Union hospital, Tongji medical college, Huazhong university of science and technology, Wuhan, China

b. Key Laboratory of Applied Surface and Colloid Chemistry, Ministry of Education, School of Chemistry and Chemical Engineering, Shaanxi Normal University, Xi'an 710119, China.

*To whom correspondence should be addressed. E–mail: entwjkong@hust.edu.cn, zhenghaoquan@snnu.edu.cn, sunyu@hust.edu.cn.

Characterization of the materials

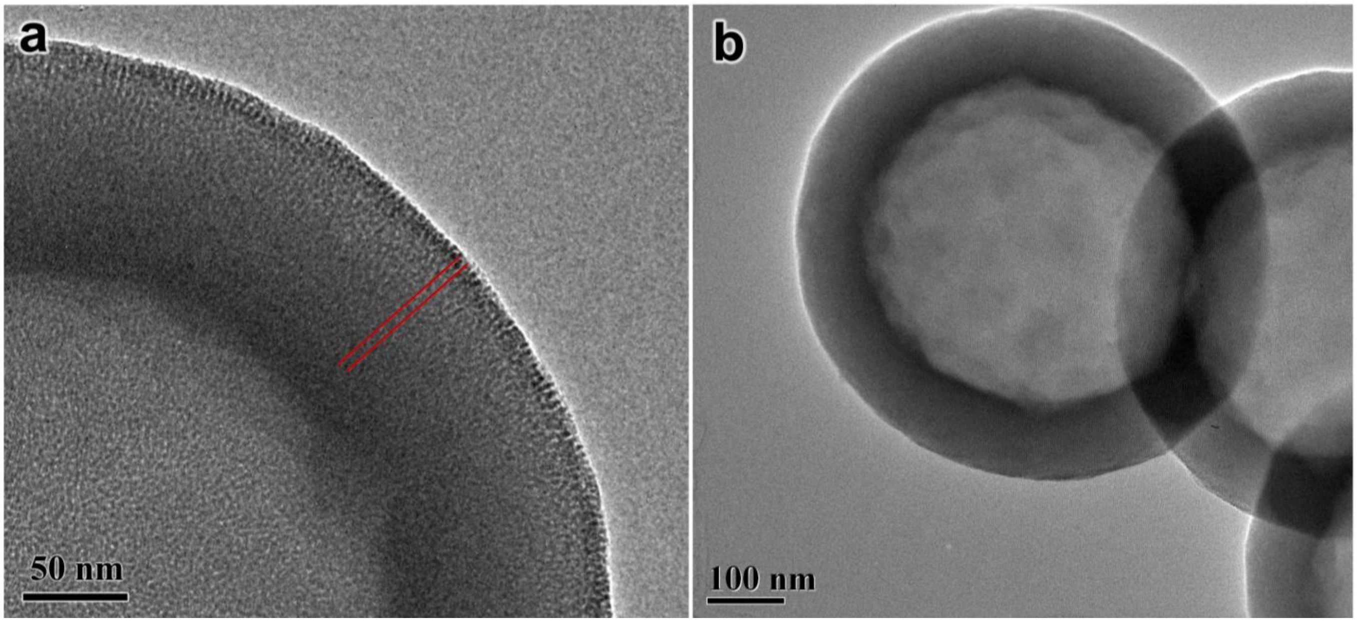

Figure. S1. The HRTEM image of HMS (a) and TEM image of GM/HMS (b).

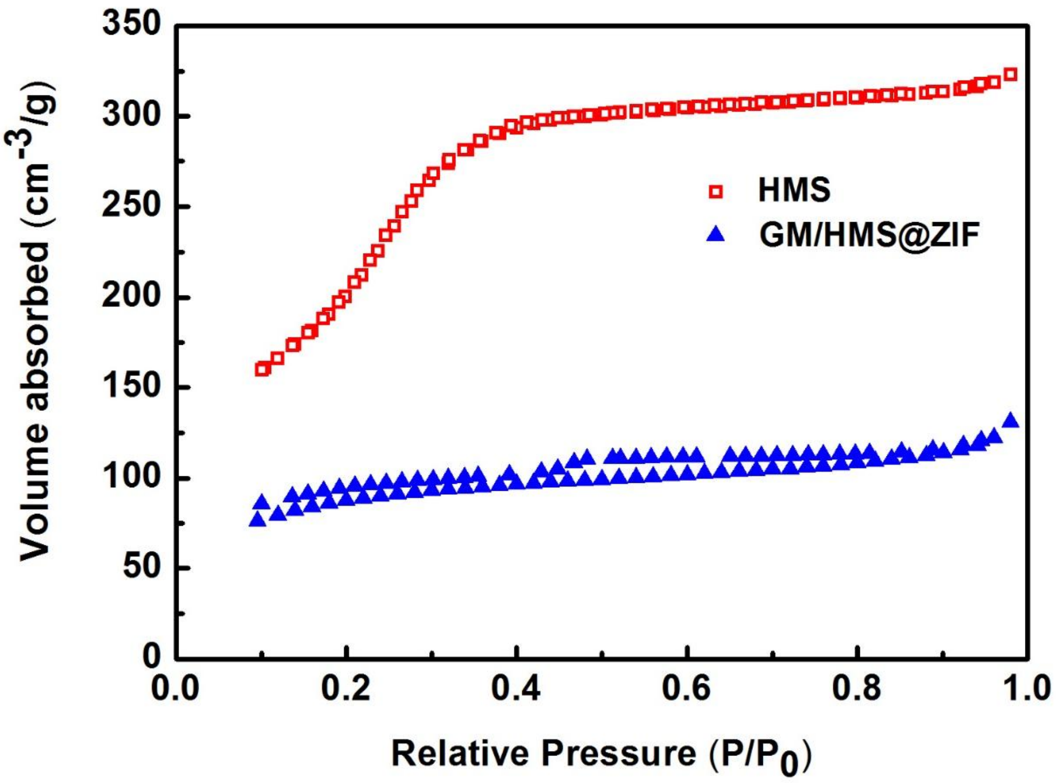

Figure S2. N_2 adsorption/desorption isotherms of HMS and GM/HMS@ZIF.

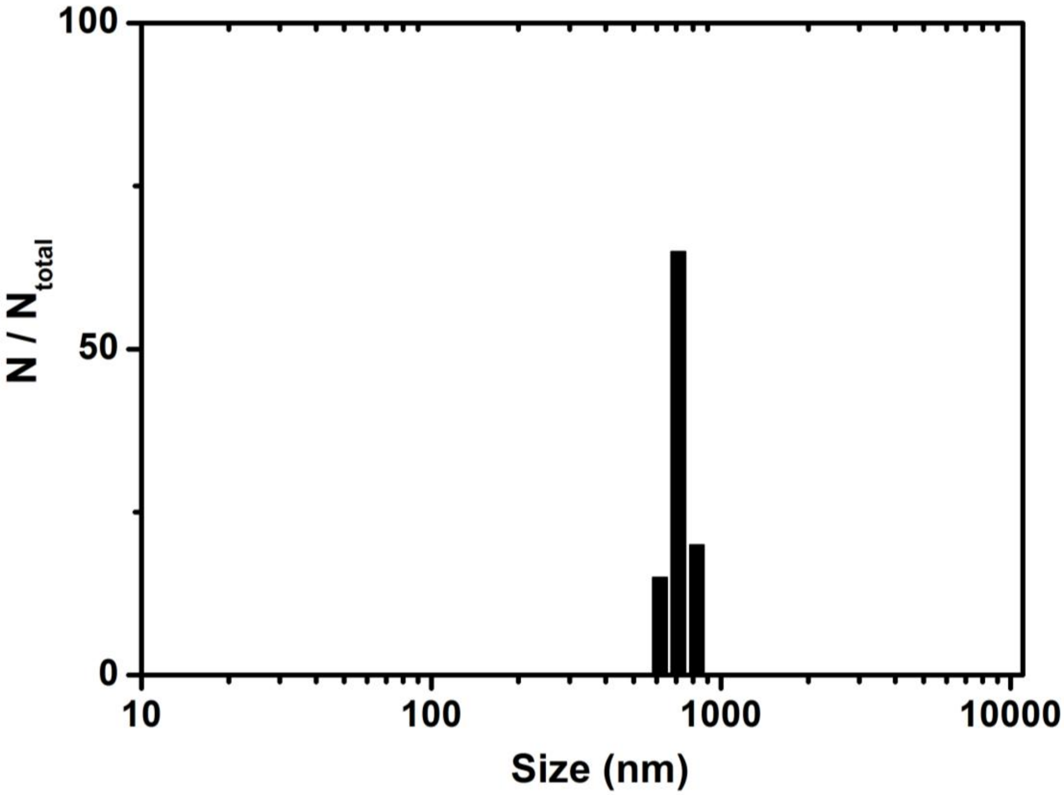

Figure S3. Size distributions of GM/HMS@ZIF in 0.9% NaCl solution.

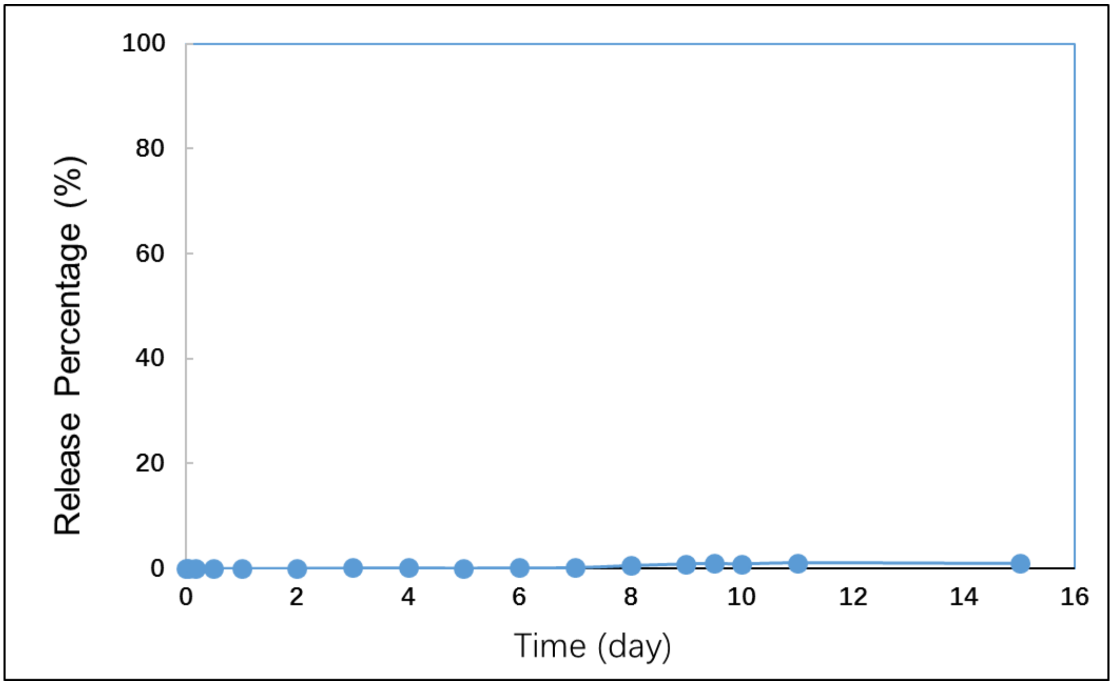

Figure S4. Release profiles of GM from GM/HMS@ZIF capsules at pH 7.4 for 15 days.

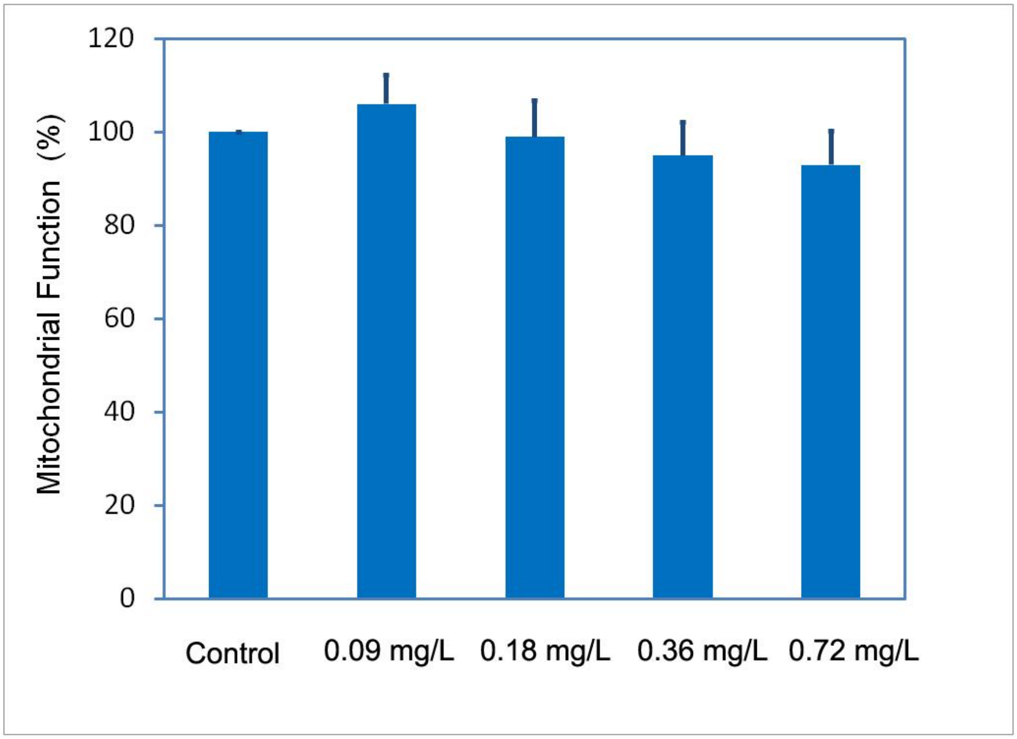

Figure S5. The function of mitochondria in the MCF-7 cells treated for 24 h.
